# Ticks are unlikely to play a role in leprosy transmission in the Comoros (East Africa) as they do not harbour *M. leprae* DNA

**DOI:** 10.3389/fmed.2023.1238914

**Published:** 2023-10-04

**Authors:** Lena Krausser, Elien Chauvaux, Magalie Van Dyck-Lippens, Amina Yssouf, Younoussa Assoumani, Pablo Tortosa, Bouke Catherine de Jong, Sofie Marijke Braet

**Affiliations:** ^1^Department of Biomedical Sciences, Institute of Tropical Medicine (ITM), Antwerp, Belgium; ^2^University of Antwerp, Antwerp, Belgium; ^3^Research Foundation Flanders (FWO), Brussels, Belgium; ^4^Unité Mixte de Recherche Processus Infectieux en Milieu Insulaire Tropical (UMR PIMIT), Université de La Réunion, CHU de La Réunion, Plateforme Technologique CYROI, Sainte-Clotilde, Réunion Island, France; ^5^Plan National de Lutte contre le Paludisme, Moroni, Comoros; ^6^Damien Foundation, Brussels, Belgium; ^7^National Tuberculosis and Leprosy Control Program, Moroni, Comoros; ^8^Université de La Réunion, Fédération de recherche Environnement, Biodiversité et Santé, Saint-Denis, Réunion Island, France

**Keywords:** leprosy, *Mycobacterium leprae*, ticks, transmission, vector, reservoir, cattle

## Abstract

**Introduction:**

Leprosy, one of the oldest known human diseases, continues to pose a global challenge for disease control due to an incomplete understanding of its transmission pathways. Ticks have been proposed as a potential contributor in leprosy transmission due to their importance as vectors for other infectious diseases.

**Methods:**

In 2010, a sampling of ticks residing on cattle was conducted on the islands Grande Comore, Anjouan, and Mohéli which constitute the Union of the Comoros where leprosy remains endemic. To investigate the potential role of ticks as a vector in transmission of leprosy disease, molecular analyses were conducted.

**Results:**

Out of the 526 ticks analysed, none were found to harbour *Mycobacterium leprae* DNA, as determined by a quantitative polymerase chain reaction (qPCR) assay targeting a family of dispersed repeats (RLEP) specific to *M. leprae*.

**Discussion:**

Therefore, our results suggest that in the Union of the Comoros, ticks are an unlikely vector for *M. leprae*.

## Introduction

Leprosy is a mutilating disease caused by the intracellular bacilli *Mycobacteria leprae* (*M. leprae*) and/or *lepromatosis* (1). Despite the World Health Organization (WHO) removing leprosy from its list of public health concerns in 2001, the lack of significant reduction in new cases and the detection of leprosy in children indicate that transmission of the disease is still ongoing (2). This is evident in regions where active measures are taken to identify cases, such as door-to-door screenings, which consistently uncover new leprosy patients. Additionally, the prevalence of severe disabilities at the time of diagnosis in many countries suggests delayed detection and diagnosis (3). As a result, it is becoming increasingly apparent that we only see the tip of the iceberg of the global leprosy burden.

The exact transmission route of leprosy has not been fully elucidated yet. Different sites have been identified as potential entry and exit for *M. leprae* bacilli to the human body, namely the nose, mouth and skin (4). The highest bacillary burden is found in the epidermis of leprosy patients (5). The most probable transmission route of leprosy is via the aerial route (6), caused by the prolonged close contact to leprosy patients. Especially multibacillary patients are considered to drive leprosy transmission, given the high bacterial load. However, the nine-banded armadillo (7, 8), red squirrels (9), and chimps (10) have been confirmed as animal reservoirs and zoonotic transmission of *M. leprae* has been confirmed by genotyping (8) infected armadillos and leprosy patients in the US. Thus, the question as to whether the transmission pathway is direct or (partially) vector-driven remains unresolved (4).

The vector competence of *Amblyomma sculptum* from the family of hard ticks (Ixodidae) for *M. leprae* was demonstrated by Ferreira et al. (11) by artificially feeding adult females with *M. leprae* Thai-53 infected rabbit blood. Transovarial transmission of *M. leprae* was confirmed by the *M. leprae* specific RLEP qPCR. These findings are supported by results of Tongluan et al. (12) who injected *Amblyomma maculatum* ticks at adult and nymph stage with an *M. leprae* Thai-53 suspension derived from infected nude mice footpads. They confirmed the presence of *M. leprae* DNA in F_1_ larvae and F_1_ nymphs via RLEP qPCR. Both studies were able to grow *M. leprae* in cell lines derived from ticks, viability was confirmed by examination of the normalized expression levels of the *M. leprae esxA* gene (12) or 16S rRNA RT-qPCR (11). Transmission of *M. leprae* to a vertebrate host followed by an infection was only shown for *M. leprae* cultivated in and isolated from tick-derived cell lines. When inoculated directly into the footpad, these bacilli are able to establish a prolonged infection in mice (11, 12). Blood-feeding tick larvae were able to transfer viable *M. leprae* to a rabbit model. However, rabbit skin was analysed already 5 days after inoculation, a time frame too short to confirm a stable infection of the vertebrate host (11). Even though these studies were mainly aiming towards being able to grow *M. leprae* bacilli *in vitro* in a cell line, the experimental data suggests that there is a possibility for a transmission route of leprosy via ticks after taking their blood meal on a person with leprosy.

The Union of the Comoros has the highest *per capita* incidence of leprosy in Africa [as high as seven cases per 10,000 individuals on Anjouan (13, 14)], making it the only country of the African continent that did not reach the elimination target of less than 1 patient/10,000 population postulated by the WHO (3, 15). Despite the persistent efforts of the National Tuberculosis and Leprosy Control Programme, including intensified screenings since 2008 and the administration of post-exposure prophylaxis within the framework of the PEOPLE and BE-PEOPLE studies (Clinicaltrials.gov: NCT03662022 and NCT05597280), leprosy, a poverty-related disease, remains endemic on the islands Anjouan and Mohéli. In contrast, the wealthiest of the three islands, Grande Comore, is not considered a leprosy endemic region. Leprosy has a long incubation period of several months to decades, with an average of 2–4 years (16), which implies ongoing transmission of the disease by the high proportion of affected children on Anjouan and Mohéli (2). The potential contribution of non-human animal and environmental reservoirs to the transmission of leprosy represents a knowledge gap towards interrupting leprosy transmission. Further, the role of ticks as biological or mechanical vector has not been confirmed by epidemiological studies yet. Therefore, this study sought to investigate the presence of *M. leprae* DNA in a tick collection obtained from the Union of the Comoros as a means of further elucidating the potential involvement of ticks as a vector in leprosy transmission.

## Materials and methods

### Samples

A total of 526 ticks from a previously described collection (17) from the Union of the Comoros were selected for screening for the presence of *M. leprae* DNA. Specimens were shipped and stored in molecular grade pure ethanol (Avantor, United States) at −20°C to the Institute of Tropical Medicine in Antwerp, Belgium. From the leprosy-endemic islands Anjouan (*n* = 134) and Mohéli (*n* = 129) 263 ticks were available. The prevalence of leprosy on Anjouan and Mohéli by the end of 2017 was 4.57/10,000 (18). A summary of leprosy prevalence per sampled district on Anjouan and Mohéli can be found in Supplementary Table S1. As a comparator, *n* = 263 ticks were selected from Grande Comore where leprosy is not endemic. All ticks were morphologically inspected and classified according to the guide by Walker et al. (19) before they were molecularly examined for the presence of *M. leprae* DNA.

### DNA extraction

One half of each tick was used for DNA extraction. The ticks were ground with a mortar and pestle in 1 mL phosphate-buffered saline. To avoid DNA contamination mortars and pestles were autoclaved, treated with bleach, and rinsed prior to use and a new set was used for each sample. Subsequently, 200 μL of the resulting suspension were incubated with 200 μL in-house lysis buffer (Tris-HCl – pH 7.5, EDTA 0.5 M pH 8, 6 M GuHCl, Tween 20, Triton X-100, diatomaceous earth) and 20 μL proteinase K solution (Promega, United States) in a shaking incubator for 1 h at 60°C and 200 rpm. The lysed suspension was further extracted with the Maxwell^®^ 16 FFPE PLUS Tissue LEV DNA purification Kit (Promega, United States), following the manufacturers’ protocol. To control for contamination throughout the extraction procedure, each run included a negative (molecular grade water) and a positive extraction control (suspension of mouse footpad infected with *M. leprae* Thai-53, BEI reference number: 19352).

### qPCR assay

To quantify *M. leprae* DNA in the tick extracts, a qPCR assay targeting a family of dispersed repeats (RLEP) (20) was used as described previously (21) for 45 cycles (positivity cut-off <40 C_q_), using the StepOnePlus^™^ qPCR cycler and StepOne software v2.3 (Applied Biosystems, United States), the primer and probe sequences and cycling conditions can be found in Supplementary Table S2. With this assay 36 out of 37 RLEP copies in the *M. leprae* genome are detected. Samples were tested in triplicate and considered positive when two of the three replicates were under the positivity cut-off. Non-template controls (molecular grade water) to control for contamination during the qPCR procedure and a gDNA (*M. leprae* NHDP, BEI reference number: 19350) standard curve for quantification with 1:10 dilutions from 3 × 10^5^ to 3 × 10^1^ RLEP copies were included in each run. An internal positive control (IPC, Eurogentec, Belgium) was spiked into each well to detect inhibition during the qPCR run.

### Statistical analysis

To determine the significance of the difference between ticks selected from the leprosy endemic (Anjouan and Mohéli) and non-endemic (Grande Comore) islands, the one-proportion z-test was applied. The significance of the sample rate ratio of ticks investigated in this study compared to the complete tick collection by Yssouf et al. (17) was calculated with the Fisher’s exact test. All statistical analyses were performed with R, version 4.3.0 for macOS (The R foundation, Vienna, Austria), the alternative hypothesis, stating significant differences between variables, was accepted at a significance level of alpha = 0.05.

## Results

### Morphological classification of ticks

Of the 263 ticks from the endemic islands of Anjouan and Mohéli, 253 (96.2%) were identified as *Rhipicephalus microplus* and 10 (3.8%) as *Amblyomma variegatum* (Table 1). The sample rate ratio analysis of species classification showed that *A. variegatum* was slightly underrepresented in the subset examined in our study with a proportion of 3.8% compared to 9.8% in the complete original collection by Yssouf et al. (17) (Supplementary Table S3).

**Table 1:** Species distribution of ticks over the three islands of the Union of the Comoros classified according to Walker et al. (19).

Group	Island	*R. microplus*		*A. variegatum*		Total	
Islands endemic for *M. leprae* transmission	Anjouan	131/134	(97.8%)	3/134	(2.2%)	134	263
	Mohéli	122/129	(94.6%)	7/129	(5.4%)	129	
Island non-endemic for *M. leprae* transmission	Grande Comore	254/263	(96.6%)	9/263	(3.4%)	263	
Total		507/526	(96.4%)	19/526	(3.6%)	526	

In our study an additional classification of the ticks by developmental stage and sex was conducted. Most of the ticks from the endemic islands were adults (*n* = 184, 70.0%), followed by ticks in the nymph stage (*n* = 77, 29.3%). Only *n* = 2 larvae (0.8%) were available for analysis (Table 2). The majority of collected ticks was identified as female (*n* = 109, 81.3% from Anjouan; *n* = 102, 79.1% from Mohéli; *n* = 167, 63.5% from Grande Comore). For a small proportion of ticks (4.6%) the sex could not be identified in our study because the determining features in some nymphs and larvae were inconclusive (Table 2).

**Table 2:** Distribution of developmental stages and sex of ticks classified and investigated in this study.

	Endemic (Anjouan + Mohéli)		Non-endemic (Grande Comore)	
Developmental stage				
Adult	184	(70.0%)	243	(92.4%)*
Nymph	77	(29.3%)	20	(7.6%)*
Larva	2	(0.8%)	0	(0.0%)
Total	263	(100%)	263	(100%)
Sex				
Female	211	(80.2%)	167	(63.5%)*
Male	43	(16.3%)	81	(30.8%)*
Undetermined	9	(3.4%)	15	(5.7%)
Total	263	(100%)	263	(100%)

^*^Proportions that are significantly different (*p* < 0.05) in the sample proportion from the non-endemic island compared to the endemic islands.

### Detection of *M. leprae* DNA by RLEP qPCR

None of the 526 tested DNA extracts from ticks resulted in a positive result in the RLEP qPCR. For none of the triplicates an amplification curve showed during the qPCR assay and therefore the positivity cut-off of 40 C_q_ was fulfilled. The limit of detection of the RLEP qPCR assay is as low as 30 RLEP copies per 2 μL added to each qPCR reaction, which correlates with approximately one *M. leprae* bacillus. All positive extraction controls resulted in a positive qPCR result. Negative extraction controls and non-template controls were negative on qPCR, indicating the absence of DNA contamination during the extractions and qPCR assays. IPC was spiked into the DNA extracts before qPCR quantification. Results were consistent within each qPCR run which confirms the absence of qPCR inhibition. A summary of the qPCR results of RLEP and IPC can be found in Supplemental File 1.

## Discussion

This study is the first to use molecular tools to screen wild, animal-derived ticks from a leprosy endemic country for the presence of *M. leprae*. The absence of *M. leprae* DNA was confirmed in all tested specimens from the Comoros. Next to *M. leprae*, *M. lepromatosis* can also cause leprosy disease in humans (1). We have tested the leprosy patient cohort in the Comoros for the presence of *M. lepromatosis* DNA by qPCR assay, with results suggesting that *M. leprae* is the only causative agent for leprosy on the Comoros (manuscript in preparation). Therefore, in this study ticks were only screened for the presence of *M. leprae* DNA.

In the search for drivers for leprosy transmission, two previous studies (11, 12) identified ticks from the genus *Amblyomma* as potential competent vectors for *M. leprae*. More specifically, under experimental conditions the transovarial transmission and the survival of *M. leprae* in female ticks and tick-derived cells was confirmed. The majority of the wild tick collection analysed in our study were adult females, which are able to harbour and transmit *M. leprae* under experimental conditions. The small proportion of nymphs, which is the developmental stage most likely to parasitize humans and transmit other tick-borne diseases such as lyme disease (22) and ehrlichiosis (23), could explain our inability to detect *M. leprae* DNA in the tick collection that was studied.

Further, the tick collection consisted of a small ratio of *Amblyomma* ticks, the species with proven capacity to harbour *M. leprae* (11, 12), compared to *R. microplus*. Only 10 out of 263 (3.8%) ticks from the endemic islands Anjouan and Mohéli were *A. variegatum* while Yssouf et al. (17) classified 73 out of 742 (9.8%) ticks as *A. variegatum*. The reason for the different species distribution is that only a subset of the original collection was available for analyses at ITM, Antwerp. The selected number of ticks from Grande Comore, used as non-endemic controls, was matched to the species distribution found for the endemic islands in this study. Accordingly, the percentage of *A. variegatum* was smaller than the one found by Yssouf et al. on this island. However, both *Rhipicephalus* and *Amblyomma* ticks belong to the family of Ixodidae (or hard ticks). In their previous studies Tongluan et al. and Ferreira et al. were able to maintain *M. leprae* in *Ixodes*-derived cell lines which suggests a similar potential of all members of the Ixodidae family as a vector for *M. leprae*.

Even though the ticks analysed in our study were collected from cattle and goats and not from humans, feeding of cattle ticks on humans seems probable in situations where humans and livestock live closely together. For both *R. microplus* and *A. variegatum* which mainly feed on cattle and other large animals (24), such cross-over events have been reported (25–27). A recent publication by Faber et al. (28) is raising the hypothesis that a skin disease in water buffaloes described as lepra bubalorum could be caused by *M. leprae* and therefore act as animal reservoir. However, evidence for cases in Indonesia is only historical as there were no further reports for lepra bubalorum in cattle since 1961 (29) and there is no water buffalo population described in the Union of the Comoros (30).

Different other vectors have been suggested for the transmission of *M. leprae*, e.g., arthropods such as mosquitos (Aedes, Culex, Rhodnius) (31–33), flies (*Musea, Calliphora and Stomoxys*) (34), and sand flies (*Phlebotomus, Sergentomyia*). The latter are unlikely vectors as they cannot maintain viable *M. leprae* bacilli (35). Early studies on mosquitos confirmed the presence of acid-fast bacilli in the proboscis of mosquitos (*A. aegypti* and *C. fatigans*) after experimentally feeding on untreated leprosy patients (31, 32). However, viability determined by fluorescence microscopy reduced within seven days after feeding (32). Da Silva Neumann et al. have investigated *R. prolixus*, *A. aegypti*, and *C. quinquefasciatus* as possible vector, with the result that only *R. prolixus* has the ability to defecate infective *M. leprae* up to 20 days after infection with *M. leprae* Thai-53 infected rabbit blood (33). Additionally, amoeba have been found to have vector potential as they can phagocytose *M. leprae*. *In vitro* experiments showed that *M. leprae* can survive up to 72 h within the *Acanthamoeba* and up to 8 months in amoebal cysts while retaining infectivity for a nude mouse model (36, 37). However, for none of these vector candidates a clear correlation with leprosy infections in humans was identified.

Even though Ixodes ticks are potential competent vectors for *M. leprae in vitro* and pathogen transmission from livestock to humans via ticks is probable, all ticks from Anjouan, Mohéli, and Grande Comore that were investigated tested negative for *M. leprae* DNA. This finding lessens the chance that leprosy is a tick-borne zoonosis in the Union of the Comoros, rather than spread by human-to-human transmission.

Our results support the hypothesis that most leprosy infections are caused by human-to-human interactions rather than by a non-human animal or environmental reservoir of *M. leprae* and that close contact to a leprosy patient is the driving force of transmission. For the definitive exclusion of the role of ticks in the transmission of leprosy disease, a larger number of ticks also from other leprosy endemic regions should be analysed. The exploration of human-derived ticks and particularly ticks parasitising leprosy patients should be the focus of such studies. Further, qualitative case control studies investigating daily activities of leprosy patients and healthy controls will be useful for the generation of new hypotheses on the driving factors of leprosy transmission.

## Data availability statement

The original contributions presented in the study are included in the article/Supplementary material, further inquiries can be directed to the corresponding author.

## Ethics statement

All ticks were manually sampled in the presence of cattle owners. No suffering was imposed to sampled animals and the disturbance associated with sampling was underneath the regulatory threshold requiring the submission of the protocol to an institutional ethics committee, as described on European Union Directive 2010/63/UE. For the study of ticks, ethical approval was not required in accordance with the national legislation and the institutional requirements.

## Author contributions

LK, EC, BJ, and SB contributed to conception and design of the study. AY and PT were involved with the tick sampling and provided the tick collection. EC and MD-L were involved in the methodology, investigation, and validation. LK, EC, and SB performed the formal analysis and statistics. SB was the project administrator and together with BJ supervised the study. LK wrote the original manuscript draft. All authors contributed to the article and approved the submitted version.
