# Supplementary material for: Ticks are unlikely to play a role in leprosy transmission in the Comoros (East Africa) as they do not harbour M. leprae DNA
Source: Front Med (Lausanne). 2023 Oct 4;10:1238914. doi: 10.3389/fmed.2023.1238914 (PMC10582737; doi:10.3389/fmed.2023.1238914)
Supplement: Supplementary file 2 [file Table_2.DOCX]

**Supplemental Table 1: Districts on Anjouan where ticks were sampled by Yssouf et al. (17) in 2010 and the leprosy prevalence per 10,000 reported by Hasker et al. (14) from those districts.**

| **District** | **Leprosy prevalence/10,000** | **Period** |
| --- | --- | --- |
| **Anjouan** |  |  |
| Pomoni | 556.7 | 2008 - 2015 |
| Domoni | 356.5 | 2007 - 2015 |
| Tsembehou | 230.6 | 2001 - 2013 |
| Mremani | 79.9 | 2014 |
| Ouani | 126.9 | 2008 - 2014 |
| Mutsamudu | 725.9 | 2001 - 2014 |
| Nioumakélé | No data available | NA |
| Sima |  |  |
| **Mohéli** |  |  |
| Fomboni | 83.6* | 2010 – 2015* |
| Wanani | 24.3* | 2010 – 2015* |
| Nioumachoua | 54.8* | 2010 – 2015* |

* Data retrieved from the National Tuberculosis and Leprosy Programme (NTLP) of the Union of the Comoros

14. Hasker E, Baco A, Younoussa A, Mzembaba A, Grillone S, Demeulenaere T, et al. Leprosy on Anjouan (Comoros): Persistent hyper-endemicity despite decades of solid control efforts. Lepr Rev. 2017;88(3):334–42.

17. Yssouf A, Lagadec E, Bakari A, Foray C, Stachurski F, Cardinale E, et al. Colonization of Grande Comore Island by a lineage of Rhipicephalus appendiculatus ticks. Parasites and Vectors. 2011;4(1):1–8.
