# Supplementary material for: Ticks are unlikely to play a role in leprosy transmission in the Comoros (East Africa) as they do not harbour M. leprae DNA
Source: Front Med (Lausanne). 2023 Oct 4;10:1238914. doi: 10.3389/fmed.2023.1238914 (PMC10582737; doi:10.3389/fmed.2023.1238914)
Supplement: Supplementary file 3 [file Table_3.DOCX]

**Supplemental Table 2: Primer and probe sequences and the respective qPCR conditions for each RT-qPCR assay.**

| **Name** | **Sequence 5’ – 3’** | **Final concentration/**  **qPCR reaction** | **qPCR conditions** | | **Reference** |
| --- | --- | --- | --- | --- | --- |
| MLRLEPTaq-F | gcagtatcgtgttagtgaa | 400 nM | 1 x  45 x | 95 °C, 10 min  95 °C, 10 sec  60 °C, 1 min | Truman et al., 2008 (21) |
| MLRLEPTaq-R | catacggcaaccttctagcg | 400 nM |  |  |  |
| MLRLEPTaq-P | tcgatgatccggccgtcggcg | 100 nM |  |  |  |

21. Truman RW, Andrews PK, Robbins NY, Adams LB, Krahenbuhl JL, Gillis TP. Enumeration of Mycobacterium leprae using real-time PCR. PLoS Negl Trop Dis. 2008;2(11).
