# Supplementary material for: Ticks are unlikely to play a role in leprosy transmission in the Comoros (East Africa) as they do not harbour M. leprae DNA
Source: Front Med (Lausanne). 2023 Oct 4;10:1238914. doi: 10.3389/fmed.2023.1238914 (PMC10582737; doi:10.3389/fmed.2023.1238914)
Supplement: Supplementary file 4 [file Table_4.DOCX]

**Supplemental Table 3: Differences in species distribution of the whole tick collection classified by Yssouf et al. (17) and the subset that was re-classified for this study. Only numbers from the endemic islands Anjouan and Mohéli are presented. The sample rate ratio was calculated with a Fisher’s exact test.**

| **Species** | **Yssouf *et al.* (17)** | | **This study** | | **Sample rate ratio (95% CI)** | |
| --- | --- | --- | --- | --- | --- | --- |
| *Rhipicephalus microplus* | 669/742 | (90.2%) | 253/263 | (96.2 %) | 1 | (ref) |
| *Amblyomma variegatum* | 73/742 | (9.8 %) | 10/263 | (3.8 %) | 0.4 | (0.2 – 0.7) |

17. Yssouf A, Lagadec E, Bakari A, Foray C, Stachurski F, Cardinale E, et al. Colonization of Grande Comore Island by a lineage of Rhipicephalus appendiculatus ticks. Parasites and Vectors. 2011;4(1):1–8.
